# European Bat Lyssavirus in Scottish Bats

**DOI:** 10.3201/eid1104.040920

**Published:** 2005-04

**Authors:** Sharon M. Brookes, James N. Aegerter, Graham C. Smith, Derek M. Healy, Tracey A. Jolliffe, Susan M. Swift, Iain J. Mackie, J. Stewart Pritchard, Paul A. Racey, Niall P. Moore, Anthony R. Fooks

**Affiliations:** * World Health Organization Collaborating Centre for the Characterisation of Rabies and Rabies-Related Viruses, Surrey, United Kingdom;; † Central Science Laboratory, York, United Kingdom;; ‡ University of Aberdeen, Aberdeen, United Kingdom;; § Scottish Natural Heritage, Perthshire, United Kingdom

**Keywords:** Lyssavirus, EBLV-2, seroprevalence, Daubenton bats, Scotland, research

## Abstract

We report the first seroprevalence study of the occurrence of specific antibodies to European bat lyssavirus type 2 (EBLV-2) in Daubenton's bats. Bats were captured from 19 sites across eastern and southern Scotland. Samples from 198 Daubenton's bats, 20 Natterer's bats, and 6 Pipistrelle's bats were tested for EBLV-2. Blood samples (N = 94) were subjected to a modified fluorescent antibody virus neutralization test to determine antibody titer. From 0.05% to 3.8% (95% confidence interval) of Daubenton's bats were seropositive. Antibodies to EBLV-2 were not detected in the 2 other species tested. Mouth swabs (N = 218) were obtained, and RNA was extracted for a reverse transcription–polymerase chain reaction (RT-PCR). The RT-PCR included pan lyssavirus-primers (N gene) and internal PCR control primers for ribosomal RNA. EBLV-2 RNA was not detected in any of the saliva samples tested, and live virus was not detected in virus isolation tests.

## Abstract 2

Daubenton bats may roost infrequently in human dwellings, so risk for human contact is low.

Rabies is a public health problem in most parts of the world. In Europe, in addition to classic carnivore-based rabies virus strains, 2 European bat lyssaviruses (EBLV-1 and EBLV-2) have been identified (>700 cases) in several European bat species (*1*). In 2003, a new bat virus, West Caucasian bat virus, was reported in Europe (*2*). Classical rabies virus is the archetype virus of the *Lyssavirus* genus that with 5 other genera make up the family *Rhabdoviridae* within the order Mononegavirales. The *Lyssavirus* genus is differentiated into 7 genetically divergent lineages, Rabies virus (genotype 1), Lagos bat virus (genotype 2), Mokola virus (genotype 3), Duvenhage virus (genotype 4), EBLV-1 (genotype 5), EBLV-2 (genotype 6), and Australian bat lyssavirus (genotype 7). With 1 exception (Mokola virus), all remaining genotypes have been isolated from bats (*3*). EBLVs are generally not transmissible to terrestrial animals other than bats (*4*), although 3 cases in humans occurred in an 18-year period, 1 case in a Stone marten (*Martes foina*), and 5 cases in sheep (*Ovis aries*) (*5*). However, underreporting occurs throughout parts of Europe, and in some circumstances rabies is confirmed without genetic typing of the virus. This underreporting was demonstrated in a confirmed case of rabies that occurred after a 15-year-old girl was bitten on the finger by a bat of unknown species in Voroshilovgrad (now Lugansk), Ukraine, in 1977 (*6*). A lyssavirus was isolated from the girl's brain, but the virus was not genetically typed (*5*).

EBLV-2 is the only lyssavirus that has been detected in the United Kingdom (*5*). Four cases of infection with this virus in England have been reported in Daubenton's bats (*Myotis Daubentonii*): a pregnant female in 1996 in Sussex (*7*), a juvenile female in 2002 and an adult male in 2003 in Lancashire (*8**,**9*), and a juvenile female in 2004 in Surrey (*10*) (Figure 1). In November 2002 in Scotland, the first human case of rabies (with suspected bat involvement) since 1902 was reported (*11*) (Figure 1). These suspected cases were all confirmed as EBLV-2 infections by laboratory diagnosis. Rabies was reported in quarantined animals and in humans with the classical form of this disease from foreign countries (*12*).

**Figure 1:**
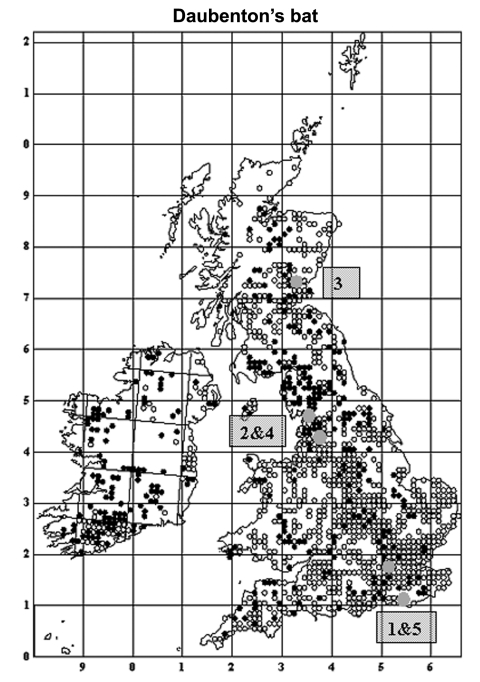
Distribution of Daubenton's bats in the United Kingdom and Ireland showing 5 cases of infection with European Bat lyssavirus type 2 (EBLV-2). Open circles are sites where Daubenton's bats were observed away from their roosts, and the closed circles are roosts of Daubenton's bats (summer and winter). The 5 numbered gray circles are sequential sites where EBLV-2–positive cases were found. Reprinted with permission of The Bat Conservation Trust (London, United Kingdom) from Distribution of Bats in Britain and Ireland 1980–1999.

The exact prevalence of EBLVs in bats in the United Kingdom is not known. From 1987 to 2004, a total of 4 of 5,030 bats were found to be infected with EBLVs in the United Kingdom through surveillance programs funded by the Department for Environment, Food and Rural Affairs. However, during this period of surveillance, only 99 Daubenton's bats were submitted for testing. Thus, the proportion of Daubenton's bats tested is underrepresented compared with estimates of Daubenton's bats in the population in the United Kingdom. Nineteen cases of infection with EBLV-2 in European bats have been documented (*5*). All (with 1 exception) were in European myotid species: Daubenton's bats and Pond bats (*M. dasycneme*). However, the latter is not indigenous to the British Isles, and Pond bat roosts have not been reported anywhere in the United Kingdom. Similar surveillance strategies have been used in other European countries (*13*).

The active surveillance described in this study investigated the prevalence of EBLV-2 in bats across southern and eastern Scotland by detecting antibodies to EBLV-2 in blood by using a modified fluorescent antibody virus neutralization (mFAVN) test and assessing oral swabs for the presence of lyssavirus RNA. The mFAVN test for EBLV-2 and polymerase chain reaction assays were developed as research techniques and used as surveillance tools after the death of a bat conservationist in 2002 (*11*). Previous studies have successfully demonstrated this approach to detect EBLV-1 in bat colonies from Spain (*14**–**16*). The emergence of new virulent bat lyssaviruses in Europe (*2*) emphasizes the need for continual appraisal and surveillance for the presence of lyssaviruses in European bats.

## Methods

### Sample Collection

**Figure 2:**
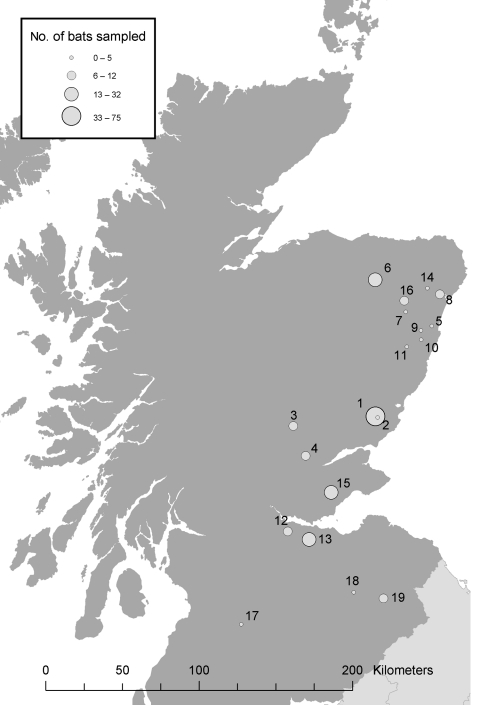
Bat sampling locations in southern and eastern Scotland. The circles indicate both the location (number) and an estimate of the number (size) of bats sampled.

From April to October 2003, a total of 229 bats were caught on 22 nights at 19 locations in Scotland (Figure 2). Each bat was assessed for health, sex, age, and reproductive status. Features examined in each bat for signs of poor health included unusual posture, matted fur, discharges from the orifices, thin appearance, excessively injured wings (rips, tears, and punctures), and excessive parasite burden. In addition, both weight and forearm measurements were taken, and bats that were unusually light for their skeletal size were examined more closely. Behavioral signs also noted included loss of coordination, seizures, and persistent aggression. Bats were required to fly at the end of sampling. An inability to fly after feeding, rehydration, and warming indicated debilitation. Any unusual observations were recorded.

A 2.9-mm, uniquely numbered bat ring (Mammal Society, London, UK) was fitted for individual recognition. Mouth swabs (saliva) were taken from each bat with either a dry sterile swab or a combination of dry and wet sterile swabs. These were stored individually in 500-µL sterile transport buffer (L15 medium [Sigma, St. Louis, MO, USA] containing 2 mmol L-glutamine, 50 µg/mL of penicillin, 2 µg/mL of streptomycin, 2 µg/mL of nystatin, and 2% fetal calf serum).

Blood (up to 100 µL) was taken by puncture from the antebrachial or uropatagial veins (in some bats after application of a local anesthetic cream [Lignocaine gel, Dunlop's Vet Supplies, Dumfries, UK]) by using a 26-gauge needle and then collected by using 10- to 50-µL heparinized glass capillary tubes (Statspin, Norwood, MA, USA). A proprietary antibleeding product (Hemablock veterinary wound powder, Dunlop's Vet Supplies) was then applied to each puncture site. The capillary tubes were then emptied into a sterile screw-topped tube. To increase blood volume obtained, 50 bats were attached by elastic bands to a thin cork board that was placed on a heated surface (≈43°C) to ensure vasodilatation of the peripheral veins. All samples were refrigerated and stored at ≈4°C until testing (1–5 days later). Capturing, handling, ringing, and sampling of bats were done under guidelines approved by the Home Office (UK Project Licenses PPL 60/3122 and PPL 30/1948).

From 25% to 35% of sampled bats were recaptured bats. Recapturing occurred within days of original sampling and also during a 1-year period from the date of initial sampling. This finding suggests that the sampling procedure was not harmful to bats. Ethical constraints in the United Kingdom restrict the resampling of wild bats within 3 months of capture. For this reason, additional sampling opportunities were limited.

### Sample Analysis

#### mFAVN Test

A fluorescent antibody test (a virus neutralization assay) is routinely used to measure levels of antibodies to rabies virus in sera from vaccinated animals by using rabies virus strain CVS. The mFAVN test used in this study was based on the routine test but used an EBLV-2 virus (RV628, a Daubenton's bat isolate from the United Kingdom in 1996, EBLV-2a GenBank U89478/AY721613) (*7*), instead of rabies virus. A conjugate (Centacor, Fujirebio Diagnostics Inc., Malvern, PA, USA) was used at a dilution of 1:40. Samples were analyzed in duplicate because of their small volume and serially diluted using a 3-fold series (representing reciprocal titers of 9, 27, 81, and 243–19,683). No significant difference was observed in a comparison of duplicate versus quadruplicate tests on rabies-vaccinated pet sera (Veterinary Laboratories Agency, Weybridge, UK, unpub. data).

This assay was monitored for reproducibility with positive controls (anti-rabies virus sera [(Office International des Epizooties, Agence Française de Sécurité Sanitaire des Aliments, Nancy, France)] and a pooled anti-EBLV-2 [inactivated RV628] serum from rabbits) and negative controls (normal pooled dog and normal pooled rabbit sera, Harlan, Loughborough, UK). A positive serum sample from an EBLV-2–infected bat was not available for full validation of this test.

The mFAVN test is a quantitative procedure requiring a threshold to separate positive from negative results. To eliminate false-positive results, studies in Spain (*16*) used a reciprocal titer of 27 as a cutoff for EBLV-1, while others (*14*) used a threshold titer of 9. We used a reciprocal titer >27 as a positive cutoff level; samples with lower titers were considered negative. When applied to pooled samples, this threshold may underestimate the actual number of EBLV–2-positive bats; with a cutoff value of 1:27, weakly positive samples might have been overlooked. Further studies during consecutive years in a longitudinal study would provide confirmatory data indicating the prevalence of EBLV-2 in Scotland.

Where necessary, samples were combined to give the minimum volume (50 µL) needed for the test. Blood was pooled only across 1 species at any given site and with samples of a similar volume, such that plasma from an individual bat contributed equally to the pooled sample. As a result of pooling and being unable to determine the number of bats that were antibody positive or negative in a pooled sample, the 95% confidence intervals (CIs) were broader than if no pooling had taken place.

#### Reverse Transcriptase–Polymerase Chain Reaction (RT-PCR)

The presence of virus can be determined directly by using an RT-PCR (*17*) that detects the RNA of all lyssavirus genotypes (including EBLV-1 and EBLV-2). The sensitivity of this RT-PCR and a hemi-nested PCR is of the order of 0.1 and 10^-3^ 50% tissue culture infectious doses of rabies virus (*18*). Similar values have been obtained for EBLVs (Veterinary Laboratories Agency, Weybridge, UK, unpub. data). The interpretation of PCR results assumes that each swab contained saliva or cells from the oral cavity. To determine this, a separate ribosomal RNA (rRNA) PCR (*18*) was conducted to detect host oral rRNA. The result for the lyssavirus RNA PCR was reported as unknown if rRNA was not detected. In contrast, if the swab was positive for rRNA, the lyssavirus PCR result was reported as either positive or negative. Methods used in this study were as previously described (*17**,**18*), with 250 µL of transport medium for the initial RNA extraction.

#### Isolation of Virus

Seronegative bats were tested by a rabies tissue culture infection test (RTCIT) only, while seropositive bats were tested by both the RTCIT and the mouse inoculation test (MIT) as previously described (*19**,**20*). The RTCIT technique was conducted by using 100 µL of transport medium per well in duplicate wells on 96-well plates. For the MIT, 4-week-old outbred CD1 mice (Charles River, Margate, UK) were injected intracranially with 40-µL samples that were antibody positive. Two mice were used per sample. The MIT was conducted according to the Home Office guidelines (UK Project License PPL 70/4867), and mice were monitored for 41 days before being humanely killed.

#### Statistical Analysis

Prevalence was calculated for sites at which we did not expect to find seropositive bats. This prevalence includes bats chosen from all Scottish sites, and a separate prevalence was calculated for site 1 (Figure 2). We anticipated that site 1 would be a location where EBLV-2–positive bats might be found because this site was the geographic region in which the suspected human exposure to EBLV-2 was reported (Figure 2) (*11*). Confidence limits were calculated as follows. An initial estimate for the proportion of bats that were seropositive for EBLV-2 was calculated with a maximum likelihood function_
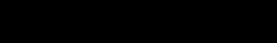
_where *p* is the unknown probability of being seropositive, *x_i_* is the number of positive sample of pools of size *i*, and *y*_i_ is the number of negative pools of size *i*. The maximum likelihood estimate (*p*^) was then used to generate the approximate 95% confidence limits by assuming 2 [ln *L* (*p*^) – ln *L* (*p*)] is approximately χ^2^ distributed. All calculations were programmed in R (*21*).

## Results

Blood was collected from 230 bats: 198 (85%) Daubenton's bats, 24 Natterer's bats (*Myotis natterii*), and 8 Pipistrelle's bats (*Pipistrellus* species). Of these, blood from 224 bats was subjected to the mFAVN test. Fifty-five (24.5%) blood samples were tested individually; the rest were combined into pools containing 2–9 samples, with most containing 3 samples. The distribution of these samples across different sites is shown in the Table. The effects of pooling samples on the performance of the mFAVN test have not been fully investigated, but no evidence suggests that a pool containing multiple seropositive bat samples shows test behavior quantitatively different from a pool containing 1 seropositive bat sample.

**Table:** Number of samples analyzed, by bat species and location*

Site	Daubenton's	Natterer's	Pipistrelle's
1†	69 (21)		6 (1)
2	0		
3	10 (3)		
4	0	12 (3)	
5	3 (3)		
6	20 (20)		
7	2 (2)		
8	6 (6)		
9	1 (1)		
10	2 (1)		
11	2 (1)		
12	5 (2)	2 (1)	
13	20 (6)	2 (1)	
14	5 (4)		
15†	32 (11)		
16	8 (4)		
17	0	4 (0)	
18	4 (3)		
19	9 (0)		
Total	198 (88)	20 (5)	6 (1)

*Values in parentheses are the number of samples (pools or single) analyzed by a modified fluorescent antibody virus neutralization test. †Sites that had positive results for antibodies to European bat lyssavirus type 2.

**Figure 3:**
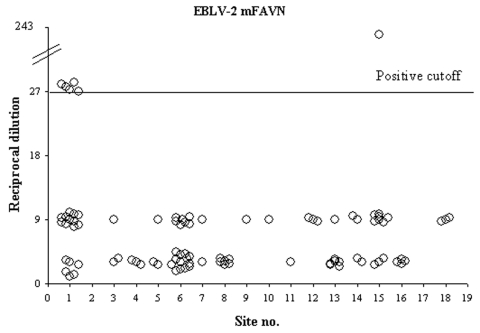
Antibody titers to European bat lyssavirus type 2 (EBLV-2) in bat sera from Scotland. An EBLV-2–specific modified fluorescent antibody virus neutralization (mFAVN) test was used to determine the level of circulating antibody in Daubenton's bats from 19 sites in Scotland. The test uses a 3-fold dilution series (9, 27, 81, 243, etc.) and the positive/negative cutoff is a titer (reciprocal dilution) of 27. Circles on the graph represent either single serum samples or pools of sera (88 for Daubenton's bats, 5 for Natterer's bats, and 1 from Pipistrelle's bats). All titers >27 are Daubenton's bats from 2 sites (5 from site 1 and 1 from site 15). No data were available for sites 2, 17, and 19.

Calculations of prevalence were performed only for blood samples (or pools) in which a successful positive or negative result was obtained. Positive samples were obtained in 4 pools (containing serum from 9, 2, 2, and 3 bats) and 2 single samples, and were exclusively from Daubenton's bats caught at 2 sites (Figure 3). This finding represents 6–18 bats since a minimum of 1 bat from each pool may have been antibody-positive. Determining whether the high value of the reciprocal titer (243) produced by 1 pool of 3 bats (site 15) (Figure 2) represents >1 seropositive bat in this pool was not possible. All Natterer's bats (5 pools) and Pipistrelle's bats (1 pool) sampled were negative. The prevalence of EBLV-2 for the Natterer's and Pipistrelle's bats tested was not significant because of the limited number of each species sampled.

Host rRNA was detected in 218 (65%) of the samples, indicating that saliva, cells, or both were present on the swab. In the remaining 35%, RNA was absent or below the limit of detection. No difference was detected in the ability to detect RNA when wet and dry swabs were compared. None of the results of the first-round or heminested PCRs with any of the samples were positive for lyssaviruses. These data suggest that none of the bats tested were actively excreting virus.

Virus isolation tests were conducted using RTCIT (all bats swabs) and MIT (antibody-positive bat swabs only). All RTCIT samples were negative and at day 41 after injection, clinical signs of infection had not developed in any mouse. These data indicate that live virus was not detectable in the oral swab samples.

If the bats tested in this study (with the exception of those at site 1) were a truly random selection of Daubenton's bats across Scotland, the likely prevalence (95% CI ) of bats testing seropositive for EBLV-2 would be 0.05%–3.80%. The 95% CI for the prevalence of EBLV-2–seropositive bats at site 1 was 2.9%–16.3%.

## Discussion

The development of techniques to detect lyssavirus infection and previous exposure is fundamental to understanding both the risks to humans posed by EBLVs and in studying virus epidemiology. Results from Spanish (*14**–**16*) and North American (*22*) studies suggest that the relationship between lyssaviruses isolated from bats, the role of the immune system, and excretion of virus in the saliva are complex. Seroprevalence levels of EBLV-1 in individual and mixed-species bat colonies in Spain during 9 years of sampling increased from 3% in 1 year to 59% the next year and subsequently decreased to 10% by the end of the study (*16*). Other investigators in the United States have reported a high seroprevalence for rabies virus (15%–20% per colony, range <5%–34%) in big brown bats (*Eptesicus fuscus*) (*22*).

Bats in the United States and Spain may coexist with rabies virus and EBLV-1, respectively; some bats are healthy and breed successfully for a number of years (*14**–**16**,**22*). If this case also is true of EBLV-2, the virus would be difficult to detect directly, but a long-term observation of antibody-positive bats would be expected.

Our study shows that the seroprevalence of EBLV-2 is generally low (<4%) and that active virus excretion is below the limit of detection used. If this prevalence of EBLV-2 was distributed evenly throughout Scotland, samples from large numbers of bats would be needed to refine this estimate. The effect of using the same detection threshold in the mFAVN test for both single and pooled samples may result in an underestimate of the number of antibody-positive bats.

Antibodies to EBLV-2 in Daubenton's bats were found at only 2 of 19 sampled sites in Scotland. Whether the lower prevalence rates in Scotland will persist and are a function of either the bat or the virus being at the northerly edge of its range, or as suggested by a Spanish study (*16*), will change considerably over time, is not known.

EBLV-2 was not detected on oral swabs of the Daubenton's bats. These data suggest that virus was not excreted by the bats at the time of sampling and that an abortive peripheral infection with sterilizing immunity to EBLV-2 may have occurred. Thus, our findings are different from those in Spain and North America in which a low proportion of bats showed measurable levels of EBLV-1 or rabies virus in saliva associated with detectable antibody titers (*14**–**16**,**22*). These data also imply that differences in virulence exist between EBLV-2, EBLV-1, and rabies virus. An experimental study of EBLVs in ferrets demonstrated that EBLV-2 was rapidly cleared with the onset of a substantial neutralizing antibody titer (*23*).

Our understanding of the biology of bats and their interaction with EBLVs is limited, and it affects our ability to fully interpret prevalence rates. The principal tool used to determine the rates of disease prevalence, the mFAVN test, directly measures the EBLV-2 antigen-specific neutralizing antibody response. Changes affecting the immune state of the bat may have implications in our ability to detect an EBLV-2 infection. The gravid state and other physiologic stress scenarios in bats may change their immune response with respect to lyssaviruses (*24*). Unfortunately, bats, in particular myotid bats, are most accessible in their hibernacula, their maternity roosts (when most are pregnant or lactating), or at swarming sites (of which few are known and include mainly male bats).

Despite such problems, we have detected antibodies to EBLV-2 in the blood of Daubenton's bats, albeit at low levels and low rates of prevalence. Data were insufficient to determine the geographic extent of the study sites, but both sites with seropositive bats are in well-watered lowland landscapes likely to support a high density of Daubenton's bats. Areas of higher bat density would normally be considered more likely to support endemic disease. The number of Daubenton's bats in the United Kingdom has been estimated at 150,000 (*25*), with ≈40,000 in Scotland (Scottish Natural Heritage, unpub. data), and colony sizes range from 10 to 200 with an average of 20 individuals (Bat Conservation Trust and Central Science Laboratory, unpub. data). The number of Daubenton's bats found across the United Kingdom has also been increasing by 4.4% per year since 1997 (Bat Conservation Trust, unpub. data). These data on the prevalence of EBLV-2 in Daubenton's bats, coupled with the first isolation of EBLV-2 in the United Kingdom in 1996 and the distribution of cases in the United Kingdom (Figure 1) (*7**–**10*), suggest that this zoonosis may be emerging in the United Kingdom and requires continuing surveillance and management (*5*).

Rabies virus can elicit a measurable antibody response after exposure, but not all exposures are lethal; some lead to an abortive infection (*26*). Although virus replication in the central nervous system was not measured in our study, virus replication can occur in this location without rabies developing in the host, mainly because of the susceptibility of the host to virus of low pathogenicity. Our data imply that bats in Scotland do not recover from infection after exposure to EBLV-2. Moreover, Daubenton's bats exhibit a low level of susceptibility to the virus and are subsequently developing an immune response after contact with EBLV-2 viral antigens.

In future studies, following up this research and successively resampling specific sites to establish disease profiles for ringed bats within this population will be important. To this end, blood samples of sufficient volume to permit individual tests must be obtained from seropositive bats over time at sites where positive cases have occurred (Figure 2) and at randomly selected locations.

The available evidence suggests that the prevalence of EBLV-2 in Daubenton's bats in Scotland is low and may be sporadic (*27*). These bats may roost less frequently in human dwellings than some other species; thus, the risk of human contact with infectious bats is low. Public health policies have been developed in the United Kingdom to further reduce exposure and potential for disease in those considered at risk. These measures include education, rabies vaccination for those working with bats, and postexposure treatment for people bitten or scratched by any bat species.
